# Reports of Maltreatment in a Children's Hospital: Evaluation of the Epidemiological Profile and Its Relationship with the Outcome in Fractures

**DOI:** 10.1055/s-0044-1785466

**Published:** 2024-04-10

**Authors:** Janaína Romão de Andrade, Vanessa Borges Platt, Michele Honicky

**Affiliations:** 1 Hospital Infantil Joana de Gusmão/SES, Florianópolis, SC, Brasil; 2 Universidade Federal de Santa Catarina, SC, Brasil

**Keywords:** child abuse, bone fractures, violence, negligence

## Abstract

**Objective** To describe suspected/confirmed cases of child maltreatment related to fractures in a pediatric hospital in southern Brazil.

**Method** Study of the Information System of Notifiable Diseases notifications and the victims' medical records between January/2016 and December/2020. Variables related to the victim, the perpetrator, the type of abuse, the presence of fractures, and their anatomical location and death were evaluated. Logistic regression was performed to identify fracture-related variables, adjusted for sex and age. The results were expressed in odds ratios and their respective 95% confidence intervals. It was considered significant *p* < 0.05.

**Results** There were 276 cases, 73 infants (26.4%), male predominance (151, 54.7%), with authorship of the mistreatment by relatives (245, 96,0%), 85 (31,5%), they presented fractures, with five deaths (1.9%). Factors related to the presence of fracture: age of the victim (less than two years old; *n* = 82; or 2.48; 95% CI: 1.45 - 4.25), having more than two aggressors involved ( *n* = 144; or 2.09; 95% CI: 1.16-3.75), the medium being traffic/automobile accident, ( *n* = 52; or 2.65; 95% CI: 1.04–6.75), consult an orthopedist ( *n* = 91; or 6.77 / 95% CI: 3.66–12.51), and the need for surgical intervention ( *n* = 15; OR 36.72; 95% CI: 8.22–164.03).

**Conclusions** The importance of suspicion, early identification of aggression, and the correct completion of notifications for activating the system of guaranteeing rights and removal of the aggressor was emphasized.

## Introduction

Violence against children has been present in humanity since primitive times and is often linked to the educational process at home, characterized by any acts, omissions, or negligence in the care provided to the child and which can result in death, emotional or physical harm, abuse or sexual exploitation. 1 2

In the United States, of 680 thousand cases of violence against children, 75.0% were related to neglect, 18.0% to physical violence, and 8.0% to sexual violence 3 4 5 6 - for every 1000 children, 9.1 suffer aggression, 4 5 only 8 out of 100 cases of physical violence in the U.S. are reported. 7 There is recidivism in up to 50.0% and, in these, the risk of death reaches 10.0%. 7

In Brazil, physical aggression in minors varies between 20.0% 8 and 35.1%. 1 In 2019, there were 159,063 complaints of mistreatment by Disque Direitos Humanos (Disque 100 [Call Human Rights]), an increase of 15.0% compared to 2018. 9 Of these 86,837 (55.0%) referred to domestic violence against children and adolescents: neglect (38.0%), psychological violence (23.0%), physical violence (21.0%), sexual violence (11.0%), exploitation of child labor (3.0%) and others (3.0%). 9

Many of these acts of violence do not leave physical marks. When present, they are associated with soft tissue injuries (more prevalent), with those affecting the head and abdomen regions being the main causes of death in this group. 6 10 Bone fractures are the second most common finding in victims of violence. 1 3 6 7 Up to about 50.0% of fracture occurrences occur in the first year of life - and a third of these types of injuries occur in those under 3 years of age. 3 6 11 They are the result of child violence. Suspicion should be advanced with caution when the lesions do not correspond to the mechanism of trauma or the declared history. 2 3 6 7 12 13

Thermal damage, unexplained soft tissue or skull injuries, rib and/or multiple fractures, delay in seeking medical attention, or any injury to a child who is not yet walking should draw the attention of the healthcare professional. 2 3 4 7 Spinal injuries are rare in children, but they can happen in victims of violence. 3 14 Children with disabilities require increased attention, as they are a risk group for violence, 6 7 10 15 are at greater risk of osteopenia than those without disabilities, which may predispose to pathological fractures, 4 differential diagnoses of maltreatment. 6 7

Orthogonal radiographs of the skull, spine, long bones, hands, and feet are relevant in cases of suspected violence. 3 6 7 12 16 17 18 However, they do not replace the anamnesis since it is necessary to confirm the compatibility of the report of the trauma mechanism with that evidenced in the image. 2 3 6 7 13 14

Age is one of the most important characteristics in distinguishing between accidental trauma and violence. An example is tibia fractures, which are highly suspected in children who are of preambulatory age and may be accidental in young children who are already walking. 3 6 7 19

The diagnosis of violence in the health sector 1 2 6 7 19 and notification to the responsible bodies avoids worse outcomes, such as emotional and physical sequelae and even death, especially in cases in which the victim suffers several aggressions over time in a chronic form. 1 6 7 10 11 19 20 21 22 23 Whereas neglect is the most frequent type of child abuse, 2 3 7 11 23 24 it is observed that the incidence of injuries in these cases can be reduced through a preventive approach with parents, guiding them to avoid accident-prone scenarios, including those typical of each age. 2 6 7 19

To monitor, identify, and even prevent cases of mistreatment of children and adolescents, since 2001, Brazil has compulsorily adopted the notification of suspected or confirmed cases that have been attended to in the establishments of the Unified Health System ([Sistema Único de Saúde] SUS). This notification should be forwarded to municipal epidemiological surveillance and a protection agency. 25 From these records, health authorities and managers can assemble the profile of those involved and their impact, 21 25 to develop relevant public policies to prevent and manage this sad aggravation.

This study, therefore, aims to describe the profile of children with maltreatment notification who present fractures and their related factors in a pediatric orthopedic reference center in Southern Brazil.

## Methods

This is a cross-sectional study, with data analysis of children's medical records (ages 0 to 15 incomplete) notified by mistreatment in the emergency room of a pediatric hospital in Santa Catarina from January 2016 to June 2020.

The notifications were selected according to the International Classification of Diseases (ICD-10), with the possibility of outcome in fractures and/or deformities of orthopedic management 7 11 26 ; availability of information in the medical records; and standardizing terms: “motorcycle accident,” “collision,” and “car/automobile accident” to “automobile accident.” The records that contained more than one item selected for the “type of violence” field were divided into two or more, allowing a more faithful analysis.

Variables related to the victim are categorized into age (age groups), sex (male or female), race (white and non-white), presence or absence of disability/disorder, and the municipality of residence (capital or other). Those related to the perpetrator: number of involved, gender (male or female), suspicion of alcohol use, bond/degree of kinship with the victim (father, mother, stepfather, stepmother, boyfriend, ex-boyfriend, brother, friends, caregiver, friend, unknown, person in an institutional relationship or others, specifying them) were grouped, generating the variable “known” and “unknown.”

Concerning violence, a typology was found (neglect, physical, psychological, suicide attempt, and others), being categorized into “neglect,” “physical,” and “others” (together with the others previously listed), and death as a result of aggression. Automobile accidents with a record in the victim's medical record of the non-use of legally provided safety devices were considered negligence.

In addition to manually verifying the notification forms, an analysis of the records in the patients' medical records was performed, aiming to investigate the outcome: “presence of fractures.” They were categorized in terms of presence (yes or no) based on the orthopedist's clinical and radiological diagnosis, topography (whether in the upper limbs, lower limbs, axial skeleton, or two or more body segments), and the need for surgical intervention.

The data were analyzed using the *Statistical Package for the Social Sciences* , version 22.0, by descriptive statistics in simple frequency and proportion. Binary logistic regression was employed, using the chi-square or Fisher's exact tests in the crude model (variables with *p* < 0,20). The selection method was used *backward* for the adjusted analysis, with results expressed in odds ratio (or) and respective confidence intervals (CI) of 95%. *p* < 0.05 was considered significant.

**Fig. 1:**
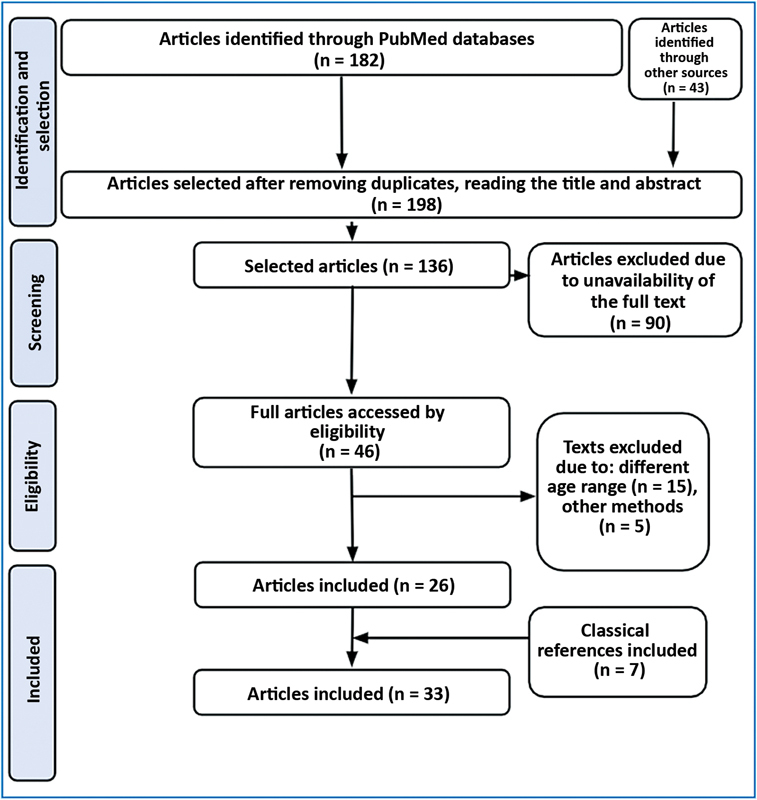
Search Strategy on Child Maltreatment With Bone Fractures, Pubmed: 2015-2020. Source: www.prisma.statement.gov [Data from the author].

In April 2020, a systematized search of the PubMed database ( *US National Library of Medicine National Institutes of Health* ) on child maltreatment yielded 182 articles ( Table 1 and Fig. 1 ).

**Table 1:** Data and search keys from the April 2020 literature review

PubMed	Child abuse x child X fractures x notification
	(“child abuse” [Mesh] OR “child abuse” OR “abused children” OR “abused child” OR “childhood abuse” OR “childhood violence” OR “violence against children” OR “violence toward children” OR “Nonaccidental Trauma in Children” OR “infant Apparent Life-Threatening Event” [Mesh] OR “Infantile Apparent Life-Threatening Event”) AND (“fractures, bone”[MeSH Terms] OR “fractures” OR “fracture”) AND (“2015/01/01”[PDAT]: “2020/12/31”[PDAT]) AND “last 5 years”[PDat] AND Humans[Mesh] AND (English[lang] OR French[lang] OR Portuguese[lang] OR Spanish[lang]) AND ((infant[MeSH] OR child[MeSH] OR adolescent[MeSH]) OR infant[MeSH:noexp] OR child, preschool[MeSH] OR infant, newborn[MeSH] OR infant[MeSH] OR adolescent[MeSH] OR child[MeSH:noexp])

Source: Prepared by the author, 2020.

The study was approved by the Institutional Research Ethics Committee (Consolidated Opinion 4.203.338/2020).

## Results

From January 2016 to December 2020, 276 notifications of suspected or confirmed cases of interpersonal or self-inflicted violence were made, corresponding to a total of 253 children and adolescents. No records confirming or excluding the diagnosis of fractures were found in 6 of the 276 cases, leaving 270 notifications for analysis.

Regarding the characteristics of children and adolescents reported as victims of maltreatment described in Table 1 , there was a predominance of males (54.7%), over 10 years of age (30.1%), of white ethnicity (89.4%), without disabilities (94.0%) and who inhabited cities that were not Florianópolis (65.2%). The age of the victim showed a significant difference according to sex. Most of the victims were boys aged 10-15 years ( *p* < 0.05).

**Table 1:** Characterization of victims of physical/self-inflicted violence (according to age, ethnicity, presence of disabilities, and the municipality of residence), according to sex, SINAN-HIJG, 2016–2020 ( *n* = *276* )

Variables	Total	Female		Male		*p* -value
	n (%)	n (%)	CI95%	n (%)	CI95%	
n	276 (100)	125 (45.3)	–	151 (54.7)	–	
** Age†** ( *n* *=* *276* )						** 0.031 b**
0-29 days	9 (3.2)	6 (4.8)	2.1–10.3	3 (2.0)	0.6–6.1	
30d – 1 year	73 (26.4)	38 (30.4)	22.9–39.1	35 (23.2)	17.1–30.7	
2 |–6 years	62 (22.5)	34 (27.2)	20.0–35.8	28 (18.5)	13.1–25.6	
6 |–10 years	49 (17.8)	19 (15.2)	9.8–22.7	30 (19.9)	14.2–27.1	
10 |–15 years	83 (30.1)	28 (22.4)	15.9–30.7	55 (36.4)	29.1–44.5	
Ethnicity* ( *n* *=* *274* )						0.994 a
White	245 (89.4)	110 (89.4)	82.5–93.8	135 (89.4)	83.3–93.4	
Not White	29 (10.6)	13 (10.5)	6.2–17.5	16 (10.6)	6.6–16.7	
Disability* ( *n* *=* *275* )						0.232 b
No	187 (94.0)	86 (96.6)	89.9–98.9	101 (91.8)	89.9–95.7	
Yes	12 (6.0)	3 (3.4)	1.1–10.1	9 (8.2)	4.2–15.1	
Municipality of residence* ( *n* *=* *273* )						0.189 a
Florianópolis	95 (34.8)	38 (30.7)	23.1–39.4	57 (38.3)	30.7–46.4	
Other ^‡^	178 (65.2)	86 (69.4)	60.6–76.9	92 (61.7)	53.6–69.3	

Abbreviations: CI95%: 95% Confidence Interval; HIJG: Hospital Infantil Joana de Gusmão; SINAN: Sistema de Informação de Agravos de Notificação.

a Chi-square test.

b Fisher's exact test.

*Data without information from all records.

†Stratification according to Marcondes 29

‡ Grouping of all Brazilian municipalities cited as the victim's residence.

The sex distribution of the perpetrators of the aggression concerning the sex of the victim was statistically significant ( *p* < 0,05). In 57.4% of the notifications, the authorship of these violences was linked to at least two suspects who acted jointly, mostly parents (50.2%), without suspicion of alcohol use in 91.3% of the notifications. Acquaintances predominated (96.0%) ( Table 2 ).

**Table 2:** Characterization of perpetrators of ill-treatment (according to the gender of the perpetrator, relationship with the victim, dichotomized relationship, number of perpetrators, and use of alcohol by the perpetrator), according to the sex of the victims, SINAN-HIJG, 2016–2020 ( *n* = *276* )

Variables	Total	Female		Male		*p* -value a
	n (%)	n (%)	CI95%	n (%)	CI95%	
**Sex** * ( *n* *=* *248)*						**0.001** a
Male	55 (22.2)	18 (15.5)	9.9–23.4	37 (28.0)	21.0–36.4	
Female	55 (22.2)	37 (31.9)	24.0–41.0	18 (13.6)	8.7–20.7	
Perpetrators of both sexes involved in the assault	138 (55.7)	61 (52.6)	43.3–61.6	77 (58.3)	49.6–66.5	
Bond with the victim* ( *n* *=* *251* )						0.865 b
Mother	39 (15.5)	22 (18.5)	12.4–26.6	17 (12.8)	8.1–19.8	
Father	25 (10.0)	12 (10.1)	5.7–17.0	13 (9.9)	5.8–16.3	
Both †	126 (50.2)	56 (47.1)	38.2–56.2	70 (53.0)	44.3–61.5	
Mother + others ^‡^	13 (5.18)	7 (5.8)	2.8–11.9	6 (4.6)	2.0–9.8	
Father + others ^‡^	1 (0.40)	0 (0)	0 -0	1 (0.8)	0.1–5.3	
Own person	14 (5.58)	8 (6.7)	3.4–13.0	6 (4.6)	2.0–9.8	
Unknown	10 (3.98)	4 (3.4)	1.2–8.7	6 (4.6)	2.0–9.8	
Acquaintance or Relative	22 (8.76)	10 (8.4)	4.5–15.0	12 (9.1)	5.2–15.4	
Father + Mother + Others	1 (0.40)	0 (0)	0 -0	1 (0.8)	0.1–5.3	
**Bond with the victim** dichotomized* (*n* *=* *251* )						0.752 b
Known	241 (96.0)	115 (96.4)	91.3–98.8	126 (95.5)	90.2–98.0	
Unknown	10 (4.0)	4 (3.4)	1.2–8.7	6 (4.6)	2.0–9.8	
Number of perpetrator* ( *n* *=* *251* )						0.403 a
1	107 (42.6)	54 (45.4)	36.6–54.5	53 (40.2)	32.0–48.8	
2 or more	144 (57.4)	65 (54.6)	45.5–63.4	79 (59.8)	51.2–68.0	
Alcohol use* ( *n* *=* *161* )						0.263 b
No	147 (91.3)	67 (88.2)	78.5–93.8	80 (94.1)	86.4–97.6	
Yes	14 (8.7)	9 (11.8)	6.1–21.5	5 (5.9)	2.4–13.6	

Abbreviations: CI95%, 95% Confidence Interval; HIJG, Hospital Infantil Joana de Gusmão; SINAN, Sistema de Informação de Agraos de Notificação.

a Chi-square test.

b Fisher's exact test.

*Data without information from all records.

† Grouping of father and mother bonds.

‡ Grouping of all ties other than “Father” or “mother” but associated with one of them.

Neglect was the most described typology (53.9%), the beating was the most common means (33.9%), resulting in fractures in 31.5% of cases, and the axial axis was the most affected anatomical segment (40.0%). Regarding the fracture site and sex of the victim, fractures only in the axial skeleton were frequent in males, and fractures in two or more segments predominated in females ( *p* < 0,05). 15 of them (17.6%) required surgical intervention. There were five deaths: two due to gunshot wounds, one due to beatings, one due to automobile accidents, and one due to falls ( Table 3 ).

**Table 3:** Typology of cases of maltreatment, its consequences, and outcome “bone fractures” according to the sex of the victims (according to the type of violence, the environment, the presence of fractures, the type of fracture, whether there was consultation with an orthopedist and whether there was a need for surgery or deaths), SINAN-HIJG, 2016–2020 ( *n* = *276* )

Variables	Total	Female		Male		*p* -value
	n (%)	n (%)	CI95%	n (%)	CI95%	
**Type** ( *n* *=* *271* )						0.352 **a**
Negligence	146 (53.9)	61 (50.0)	41.1–58.9	85 (57.0)	48.9–64.8	
Physics	106 (39.1)	50 (41.0)	32.5–50.0	56 (37.6)	30.1–45.7	
Other**	19 (7.0)	11 (9.0)	5.0–15.7	8 (5.4)	2.7–10.4	
**Means/Instrument** * ( *n* *=* *248* )						0.297 **b**
Spanking	84 (33.9)	39 (34.8)	26.5–44.2	45 (33.1)	25.6–41.5	
Traffic Accident	52 (21.0)	21 (18.8)	12.5–27.2	31 (22.8)	16.4–30.7	
Electric Shock	33 (13.3)	14 (12.5)	7.5–20.1	19 (14.0)	9.0–21.0	
Falls	32 (12.9)	20 (17.9)	11.7–26.2	12 (8.8)	5.0–15.0	
Firearm	17 (6.9)	5 (4.5)	1.8–10.4	12 (8.8)	5.0–15.0	
Other***	30 (12.1)	13 (11.6)	6.8–19.1	17 (12.5)	7.9–19.3	
**Presence of Fractures** * ( *n* *=* *270* )						0.537 **a**
No	185 (68.5)	88 (70.4)	61.7–77.8	97 (66.9)	58.7–74.1	
Yes	85 (31.5)	37 (29.6)	22.2–38.3	48 (33.1)	25.8–41.2	
Type of fracture* ( *n* *=* *85* )						** 0.017 a**
Upper Member(s) Only	15 (17.7)	8 (21.6)	10.8–38.5	7 (14.6)	6.9–28.2	
Lower Member(s) Only	16 (18.8)	7 (18.9)	8.9–35.6	9 (18.8)	9.8–32.9	
Axial Skeleton Only	34 (40.0)	8 (21.6)	10.8–38.5	26 (54.17)	39.6–68.0	
In 2 or more segments	20 (23.5)	14 (37.8)	23.3–54.99	6 (12.5)	5.5–25.8	
**Orthopedic consultation** *(n = 276)*						** 0.024 a**
No	185 (67.0)	75 (60.0)	51.1–68.3	110 (72.9)	65.1–79.4	
Yes	91 (33.0)	50 (40.0)	31.7–48.9	41 (27.2)	21.0–34.9	
**Surgery** *( *n* *=* *276* )						0.912 **a**
No	261 (94.6)	118 (94.4)	88.6–97.3	143 (94.7)	89.7–97.3	
Yes	15 (5.4)	7 (5.6)	2.7–11.4	8 (5.3)	2.7–10.3	
**Death** *( *n* *=* *270* )						0.380 **b**
No	265 (98.1)	122 (99.2)	94.3–99.9	143 (97.3)	92.9–99.0	
Yes	5 (1.9)	1 (0.8)	0.1–6.0	4 (2.7)	1.0–7.1	

CI95% = 95% Confidence Interval%;

a Chi-square test.

b Fisher's exact test.

*Data without information from all records; NA: not applicable.

**: Moral violation, ill-treatment, suicide attempt.

***: Suicide attempt, blunt object, sharp object, threat, hanging, exogenous intoxication, evasion, “shaken baby,” burning hot object.

HIJG: Hospital Infantil Joana De Gusmão; LM or LMS: lower member / lower members; UM or UMS: upper member / upper members; NDIS: Notifiable Diseases Information System.

In the analysis adjusted for sex and age, age of the victim (less than two years) (or 2.48; 95% CI: 1.45 - 4.25), involvement of two or more aggressors (or 2.09; 95% CI: 1.16 - 3.75), the means being traffic/automobile accident, (or 2.65; 95% CI: 1.04–6.75), presence of consultation with orthopedist (or 6.77; 95% CI: 3.66–12.51), and the need for surgical intervention (or 36.72; 95% CI: 8.22–164.03) were statistically significantly associated with increased risk of fractures ( Table 4 ).

**Table 4:** Factors associated with the outcome “bone fractures” in victims of maltreatment, SINAN-HIJG, 2016–2020 ( *n* = *270* ).

	Not adjusted		Adjusted for age and gender	
Variables	CR (CI95%)	*p* -value	CR (CI95%)	*p* -value
**Sex of the victim** *(n = 270)*				
Female (125)	1	0.537	1	
Male (145)	1.18 (0.70–1.97)		1.34 (0.79–2.29)	0.283
**Age of victim** *(n = 270)*				
≥ 2 years (82)	1		1	
< 2 years (188)	2.37 (1.39–4.03)	**0.001**	2.48 (1.45–4.25)	**0.001**
**Ethnicity** *(n = 270)*				
Not white (41)	1		1	
White (239)	1.52 (0.62–3.71)	0.356	1.33 (0.53–3.31)	0.542
**Disability** *(n = 195)*				
No (186)	1		1	
Sim (9)	0.66 (0.13–3.29)	0.616	0.74 (0.14–3.81)	0.718
**Municipality** ( *n = 267* )				
Florianópolis (94)	1		1	
Other** (173)	0.33 (0.44–1.32)	0.325	0.78 (0.44–1.38)	0.395
**Gender of the perpetrator(s)** * *(n = 242)*				
Female (120)	1		1	
Male (122)	1.17 (0.67–2.03)	0.589	1.20 (0.66–2.17)	0.554
**Gender of the perpetrator(s)** * *(n = 242)*				
Female (53)	1		1	
Both (135)	1.64 (0.80–3.37)	0.176	1.68 (0.80–3.52)	0.169
Male (54)	0.79 (0.32–1.96)	0.607	0.87 (0.34–2.25)	0,775
**Number of perpetrator** *(n = 251)*				
1 aggressor (107)	1		1	
2 more aggressors (144)	2.14 (1.19–3.83)	**0.011**	2.09 (1.16–3.75)	**0.014**
**Use of alcohol by the perpetrator** ( *n= 161* )				
No (147)	1		1	
Yes (14)	0.99 (0.29–3.33)	0.988	1.26 (0.36–4.40)	0.714
**Bond with the victim** *(n = 246)*				
Unknown (10)	1		1	
Known (236)	1.72 (0.36–8.31)	0.499	1.61 (0.33–7.83)	0.554
**Typology** *(n = 265)*				
Other (18)	1		1	
Negligence (145)	3.26 (0.72–14.81)	0.126	2.60 (0.56–12.02)	0.221
Physics (102)	4.95 (1.08–22.72)	**0.040**	3.67 (0.78–17.16)	0.099
**Means** ( *n = 248* )				
***Other (53)	1		1	
Beating (79)	2.07 (0.87–4.92)	0.099	1.90 (0.78–4.60)	0.157
Traffic (51)	2.51 (0.99–6.30)	0.051	2.65 (1.04–6.75)	0.042
Shock (33)	0.13 (0.16–1.10)	0.061	0.13 (0.15–1.07)	0.057
Fall (32)	2.21 (0.79–6.19)	0.131	2.28 (0.78–6.57)	0.129
**Orthopedic Consultation** *(n = 270)*				
No (179)	1		1	
Yes (91)	5.02 (2.88–8.73)	**<0.001**	6.77 (3.66–12.51)	**<0.001**
**Surgical Intervention** *(n = 270)*				
No (255)	1		1	
Yes (15)	16.52 (3.63–75.05)	**<0.001**	36.72 (8.22–164.03)	**<0.001**
**Development: Death** ( *n= 266* )				
No (261)	1		1	
Sim (5)	1.46 (0.24–8.88)	0.684	1.09 (0.17–6.95)	0.928

Abbreviations: CI95% = 95% Confidence Interval; CR, Chance of Ratio; SC: Santa Catarina; SINAN: Sistema de Informação de Agravos de Notificação.

*Data without information from all records.

**: Municipalities that are not Florianópolis.

***: Suicide attempt, blunt object, sharp object, threat, hanging, exogenous intoxication, evasion, “shaken baby,” burning hot object.

## Discussion

Male victims, as noted in the literature, 12 were the most affected (54.7%), with a 1.2-fold risk of presenting associated fractures, when compared to females.

The extremes of age (< 2 years and > 10 years) were the groups most likely to suffer aggression. Infants (< 2 years) presented a 2.4 times higher risk of fractures when compared to those older than two years, regardless of sex, corroborating the international literature 6 7 19 and differing from that computed by Disque 100 (Dial 100), where school victims were the most listed. 9 It should be noted that the smaller the child, the more dependent on care the child is, including reaching a health service, with underreporting due to omission of care. 2

In the southern region of the country, white ethnicity predominates, 8 26 explaining the disparity between reports of violence in people of this ethnicity (89.4%) in relation to the others, different from the data of the Disque 100, which indicates the brown population as the most affected by mistreatment, followed by white and black. 9 In this study, having white skin color was associated with a 1.5 times higher risk for fractures related to abuse when compared to other skin colors.

The presence of victims with disabilities was not significant (6.0%), diverging from the literature, 1 2 7 9 15 19 leading to infer that the failure to seek emergency care for these patients could be related to possible notification errors, the lack of diagnosis of aggression and/or notification and the inability to verbalize victims with disabilities. 2 26 In this group, there was no association with fracture risk.

The greater number of notifications from other municipalities (65.2%) than the capital can be justified because the hospital is large and a reference in orthopedics in the State.

Regarding the authorship of the maltreatment, most had at least two aggressors, mainly the father and mother, simultaneously, corroborating with the literature, which maintains the pattern of parents as the main suspects of child maltreatment. 1 2 3 4 5 6 7 9 15 17 19 In 91.0% of the cases, the authors of the aggressions were not under suspicion of alcohol use, in line with the literature, 2 9 15 there was no relationship between the use of alcohol by the aggressors and the outcome fractures.

Corroborating national and international research, 2 3 4 5 6 12 neglect (53.9%) and physical aggression (39.1%) were the most prevalent typologies, with similar distribution between genders. The high prevalence of the latter may be related to the use of physical force as a form of “education” or disciplinary practice, 1 2 12 where parents who were raised through punishment and physical punishment perpetrate this culturally accepted habit, 1 2 regardless of the existence of public policies, such as Law No. 13,010–“Lei do Menino Bernardo” (Bernardo Boy Law) 27 or the Statute of the Child and Adolescent. 28

The increased risk of these two forms of maltreatment in the occurrence of fractures was 3.3 and 5.0 times higher, respectively, when compared to all the others studied. Understanding the importance of vigilance of parents to their children and especially continuing education about accidents and unintentional injuries. 2 3 7

When the means of aggression were evaluated, “traffic accidents” and” beating” responded to more than half of the notifications (54.9%), followed less expressively by electric shock (13.3%), falls (12.9%), and “others,” similar to the literature, 2 6 7 9 19 signaling for the practice of physical force as an educational disciplinary measure 1 2 12 and the absence of observance of safe transportation. 2 29 “Beating” and “traffic accidents” were related to a 2.1-and 2.5-fold risk of causing fractures.

The outcome “fractures” was 2.2 times more frequently observed in the “falls,” paying attention to the need for surveillance and supervision, especially of minors who are starting to walk. 2 3 7 Its prevalence (31.5%) was 4.5 times higher than in another national study. 15

Regarding the anatomical location of the fractures, 40.0% were present only in the axial skeleton, including the skull, a region related to a more severe outcome. 2 3 4 5 6 7 12 15 18 19 23.5% of the victims presented injuries in more than one segment, being a risk factor for mistreatment 2 3 4 5 6 7 11 18 19 , that is, a child with multiple fractures, especially in more than one anatomical site, should be evaluated more closely for this diagnostic suspicion.

Of the patients studied, regardless of gender, children under two years of age had a 2.5 times higher risk of suffering fractures than other age groups, in addition to a 1.7 times higher risk for the same outcome if the violence was perpetrated by acquaintances when compared to those who were assaulted by strangers. Other factors associated with a higher risk of fractures were aggression being committed by two perpetrators or more, victims of automobile accidents, and care provided by an orthopedist—risks respectively 2.1, 2.7, and 6.8 times higher. Considering that automobile accidents are sometimes related to negligence, a great challenge emerges: Safe transportation for children must be established in the safety rules for the transport of children in vehicles. 29

Fifteen patients (17.7%) underwent orthopedic surgery as part of the treatment, and the presence of bone fracture was related to 16.5 times more need for surgical intervention and 1.5 times the risk of death when compared to the absence of it in the study.

These data reinforce the importance of prevention, 2 29 of attention to the signs that may raise suspicions of mistreatment, and the appropriate investigation by the “front line” professional and referral to the specialist when appropriate. 6 7 19

The secondary source of the data is cited as a probable limitation, which was resolved by manually checking the notification forms one by one and checking the victim's hospital records.

## Conclusions

Due to the dependence and vulnerability inherent to the life cycle, children are a risk group for various violence, whether accidental or intentional, requiring both family education for prevention and the attention of the assistance professional in the identification and correct notification of this aggravation, with adequate management of cases, avoiding serious outcomes.
