# Supplementary material for: Reports of Maltreatment in a Children's Hospital: Evaluation of the Epidemiological Profile and Its Relationship with the Outcome in Fractures
Source: Rev Bras Ortop (Sao Paulo). 2024 Apr 10;59(2):e269–77. doi: 10.1055/s-0044-1785466 (PMC11006522; doi:10.1055/s-0044-1785466)
Supplement: Related texts

# Research article: Notificações de maus-tratos em um hospital infantil: Avaliação do perfil epidemiológico e sua relação com o desfecho em fraturas

**Keywords:** maus-tratos infantis, fraturas ósseas, violência, negligência

## Abstract

**Objetivo** Descrever os casos suspeitos/confirmados de maus-tratos infantis relacionados a fraturas, atendidos em um hospital pediátrico no Sul do Brasil.

**Método** Estudo das notificações do Sistema de Informação de Agravos de Notificação e dos prontuários das vítimas, entre janeiro/2016 e dezembro/2020. Avaliadas variáveis relacionadas à vítima, ao autor, à tipologia de maus-tratos, à presença de fraturas e sua localização anatômica e óbito. Regressão logística foi realizada para identificar as variáveis relacionadas à fratura, ajustada para sexo e idade, expressa em razão de chance e seus respectivos intervalos de confiança de 95%. Foi considerado significativo *p* < 0.05.

**Resultados** Houve 276 casos, 73 lactentes (26,4%), predomínio do sexo masculino (151, 54,7%), com autoria dos maus-tratos por parentes (245, 96,0%), 85 (31,5%), apresentaram fraturas, sendo cinco óbitos (1,9%). Fatores relacionados à presença de fratura: idade da vítima (menor de dois anos; *n* = 82; RC 2,48; IC 95%: 1,45 - 4,25), ter mais de dois agressores envolvidos ( *n* = 144; RC 2,09; IC 95%: 1,16 - 3,75), o meio ser trânsito/acidente automobilístico, ( *n* = 52; RC 2,65; IC 95%: 1,04–6,75), consultar com ortopedista ( *n* = 91; RC 6,77 / IC 95%: 3,66–12,51), e necessidade de intervenção cirúrgica ( *n* = 15; RC 36,72; IC 95%: 8,22–164,03).

**Conclusões** Ressaltada a importância da suspeição e identificação precoce da agressão, o preenchimento correto das notificações, para o acionamento do sistema de garantia de direitos e afastamento do agressor.

## Introdução

A violência contra a criança está presente na humanidade desde os tempos primitivos sendo frequentemente vinculada ao processo educativo no domicílio, caracterizada por quaisquer atos, omissões ou negligências aos cuidados prestados à criança e que podem resultar em óbito, dano emocional, físico, abuso ou exploração sexual. 1 2

Nos Estados Unidos (EUA), de 680 mil casos de violência contra crianças, 75,0% relacionaram-se à negligência, 18,0% à violência física e 8,0% à violência sexual 3 4 5 6 - a cada 1000 crianças, 9,1 sofrem agressão, 4 5 somente 8 em 100 casos de violência física nos EUA são notificados. 7 Há reincidência em até 50,0% e, nessas, o risco de morte chega a 10,0%. 7

No Brasil a agressão física em menores varia entre 20,0% 8 e 35,1%. 1 Em 2019 houve 159.063 denúncias de maus-tratos pelo Disque Direitos Humanos (Disque 100), com aumento de 15,0% em relação a 2018. 9 Dessas 86.837 (55,0%) referiam-se à violência doméstica contra crianças e adolescentes: negligência (38,0%), violências psicológicas (23,0%), física (21,0%), sexual (11,0%), exploração do trabalho infantil (3,0%) e outras (3,0%). 9

Muitas dessas violências não deixam marcas físicas e quando presentes, associam-se a lesões dos tecidos moles (mais prevalentes), sendo às que acometem as regiões da cabeça e do abdômen as principais causas de morte nesse grupo. 6 10 Fraturas ósseas são o segundo achado mais comum em vítimas de violência. 1 3 6 7 Até cerca de 50,0% das ocorrências de fraturas no primeiro ano de vida - e um terço desses tipos de lesões nos menores de 3 anos. 3 6 11 são decorrentes de violência infantil. A suspeita deve ser aventada com cautela quando as lesões não correspondem ao mecanismo de trauma ou à história declarada. 2 3 6 7 12 13

Danos térmicos, lesões inexplicáveis de tecidos moles ou no crânio, fraturas nas costelas e/ou múltiplas, atraso na busca de atendimento médico ou qualquer lesão em uma criança que ainda não anda, devem chamar a atenção do profissional de saúde. 2 3 4 7 Lesões na coluna são raras em crianças, mas que podem acontecer em vítimas de violência. 3 14 Crianças com deficiências requerem atenção redobrada, pois são grupo de risco para violência, 6 7 10 15 estão em maior risco de sofrerem osteopenia do que às sem deficiências, o que pode predispor a fraturas patológicas, 4 sendo um diagnóstico diferencial de maus-tratos. 6 7

Radiografias ortogonais do crânio, coluna vertebral, ossos longos, mãos e pés são relevantes em casos de suspeita de violência, 3 6 7 12 16 17 18 porém não substituem a anamnese, uma vez ser necessário confirmar a compatibilidade do relato do mecanismo de trauma com o evidenciado na imagem. 2 3 6 7 13 14

A idade é uma das características mais importantes na distinção entre trauma acidental e violência. Um exemplo são fraturas de tíbia, altamente suspeitas em crianças que estão na idade pré deambulatória, podendo ser acidentais em crianças pequenas que já andam. 3 6 7 19

O diagnóstico da violência no setor saúde 1 2 6 7 19 e a notificação aos órgãos responsáveis evitam piores desfechos, como sequelas emocionais, físicas e até óbito, principalmente em casos nos quais a vítima sofre diversas agressões ao longo do tempo de forma crônica. 1 6 7 10 11 19 20 21 22 23 Considerando que a negligência é o tipo mais frequente de maus-tratos infantis, 2 3 7 11 23 24 observa-se que a incidência de lesões nesses casos pode ser reduzida por meio da abordagem preventiva junto aos pais, orientando evitar cenários propensos a acidentes, incluindo os típicos de cada idade. 2 6 7 19

Visando monitorar, identificar e até prevenir os casos de maus-tratos contra crianças e adolescentes, o Brasil adota desde 2001 - de forma compulsória - a notificação de casos suspeitos ou confirmados que tenham sido atendidas nos estabelecimentos do Sistema Único de Saúde (SUS), devendo essa notificação ser encaminhada à vigilância epidemiológica municipal e a um órgão de proteção. 25 A partir desses registros, autoridades e gestores da saúde podem montar o perfil dos envolvidos e seu impacto, 21 25 de forma a desenvolver políticas públicas pertinentes na prevenção e condução deste triste agravo.

Em vista disso, esse estudo objetiva descrever o perfil das crianças com notificação de maus-tratos, que apresentam fraturas e seus fatores relacionados, em um centro de referência de ortopedia pediátrica no Sul do país.

## Método

Estudo transversal, com análise de dados de prontuários de crianças (0 a 15 anos incompletos), notificados por maus-tratos na emergência de um hospital pediátrico em Santa Catarina, de janeiro de 2016 a junho de 2020.

As notificações foram selecionadas conforme Classificação Internacional de Doenças (CID-10), com possibilidade de desfecho em fraturas e/ou deformidades de manejo ortopédico 7 11 26 ; e disponibilidade de informação nos prontuários; uniformizando-se termos: “acidente de moto,” “atropelamento” e “acidente de carro/automobilístico” para “acidente automobilístico.” As fichas que continham mais de um item selecionado no campo “tipo de violência” foram desmembradas em duas ou mais, possibilitando análise mais fiel.

Variáveis relacionadas à vítima categorizadas em: idade (faixas etárias), sexo (masculino ou feminino); raça (branca e não branca), presença ou não de deficiência/transtorno, e município de residência (capital ou outro). Às relacionadas ao autor: número de envolvidos, sexo (masculino ou feminino); suspeita do uso de álcool; vínculo/grau de parentesco com a vítima (pai, mãe, padrasto, madrasta, namorado, ex-namorado, irmão, amigos, cuidador, amigo, desconhecido, pessoa em relação institucional ou outros, especificando-os) - foram agrupadas, gerando a variável “conhecido” e “desconhecido.”

Em relação à violência averiguou-se tipologia (negligência, física, psicológica, tentativa de suicídio e outros), sendo categorizadas em “negligência,” “física” e “outras” (junção das demais listadas anteriormente), e óbito em decorrência da agressão. Acidentes automobilísticos onde houve o registro no prontuário da vítima do não uso de dispositivos de segurança legalmente previstos, foram considerados negligência.

Adicionalmente à checagem manual das fichas de notificação, foi realizada análise dos registros nos prontuários dos pacientes, objetivando investigar o desfecho: “presença de fraturas.” Elas foram categorizadas quanto à presença (sim ou não) a partir do diagnóstico clínico do ortopedista e radiológico; à topografia (se nos membros superiores, nos inferiores, no esqueleto axial ou em dois ou mais segmentos corporais) e à necessidade de intervenção cirúrgica.

Os dados foram analisados utilizando o *Statistical Package for the Social Sciences* ; versão 22.0, por estatística descritiva em frequência simples e proporção. Regressão logística binária foi empregada, utilizando os testes de qui-quadrado ou Exato de Fisher no modelo bruto (variáveis com *p* < 0,20). Para a análise ajustada, utilizou-se o método de seleção *backward* , com resultados expressos em razão de chances (RC) e respectivos Intervalos de Confiança (IC) de 95%. *p* < 0,05 foi considerado significativo.

Busca sistematizada na base de dados do PubMed ( *US National Library of Medicine National Institutes of Health* ) sobre maus-tratos infantis foi realizada em abril/2020 - 182 artigos ( Quadro 1 e Fig. 1 ).

**Quadro 1:** Dados e chaves de busca da revisão da literatura de abril de 2020

PubMed	Abuso infantil x criança x fraturas x notificação
	(“child abuse”[Mesh] OR “child abuse” OR “abused children” OR “abused child” OR “childhood abuse” OR “childhood violence” OR “violence against children” OR “violence toward children” OR “Nonaccidental Trauma in Children” OR “Infantile Apparent Life-Threatening Event”[Mesh] OR “Infantile Apparent Life-Threatening Event”) AND (“fractures, bone”[MeSH Terms] OR “fractures” OR “fracture”) AND (“2015/01/01”[PDAT]: “2020/12/31”[PDAT]) AND “last 5 years”[PDat] AND Humans[Mesh] AND (English[lang] OR French[lang] OR Portuguese[lang] OR Spanish[lang]) AND ((infant[MeSH] OR child[MeSH] OR adolescent[MeSH]) OR infant[MeSH:noexp] OR child, preschool[MeSH] OR infant, newborn[MeSH] OR infant[MeSH] OR adolescent[MeSH] OR child[MeSH:noexp])

Fonte: elaborado pela autora, 2020.

**Fig. 1:** Estratégia de busca sobre maus-tratos infantis com fraturas ósseas, Pubmed: 2015–2020. Fonte: www.prisma.statement.gov , com dados da autora.

O estudo foi aprovado pelo Comitê de Ética em Pesquisa Institucional (Parecer Consubstanciado 4.203.338/2020).

## Resultados

No período de janeiro de 2016 a dezembro de 2020 foram feitas 276 notificações de casos suspeitos ou confirmados de violência interpessoal ou autoprovocada, correspondendo a totalidade de 253 de crianças e adolescentes. Em 6 dos 276 casos não havia registros que confirmassem ou excluíssem o diagnóstico de fraturas, ficando 270 notificações para análise.

Em relação às características das crianças e adolescentes notificados como vítimas de maus-tratos descritas na Tabela 1 , houve predomínio do sexo masculino (54,7%), maiores de 10 anos de idade (30,1%), de etnia branca (89,4%), sem deficiências (94,0%) e que habitavam cidades que não eram Florianópolis (65,2%). A idade da vítima apresentou diferença significativa de acordo com o sexo, a maioria das vítimas foram meninos com idade entre 10–15 anos ( *p* < 0.05).

**Tabela 1:** Caracterização das vítimas de violência física/autoprovocada (segundo idade, etnia, presença de deficiências e município de residência), de acordo com o sexo, SINAN-HIJG, 2016–2020 ( *n* = *276* )

Variáveis	Total	Feminino		Masculino		*p* -valor
	n (%)	n (%)	IC95%	n (%)	IC95%	
n	276 (100)	125 (45,3)	–	151(54,7)	–	
** Idade†** ( *n* *=* *276* )						** 0,031 b**
0–29 dias	9 (3,2)	6 (4,8)	2,1–10,3	3 (2,0)	0,6 - 6,1	
30d – 1 ano	73 (26,4)	38 (30,4)	22,9 - 39,1	35 (23,2)	17,1 - 30,7	
2 |–6 anos	62 (22,5)	34 (27,2)	20,0 - 35,8	28 (18,5)	13,1 - 25,6	
6 |–10 anos	49 (17,8)	19 (15,2)	9,8 - 22,7	30 (19,9)	14,2 - 27,1	
10 |–15 anos	83 (30,1)	28 (22,4)	15,9 - 30,7	55 (36,4)	29,1 - 44,5	
Etnia* ( *n* *=* *274* )						0,994 a
Branca	245 (89,4)	110 (89,4)	82,5 - 93,8	135 (89,4)	83,3 - 93.4	
Não Branca	29 (10,6)	13 (10,5)	6,2 - 17,5	16 (10,6)	6,6 - 16,7	
Deficiência* ( *n* *=* *275* )						0,232 b
Não	187 (94,0)	86 (96,6)	89,9 - 98,9	101 (91,8)	89,9 - 95,7	
Sim	12 (6,0)	3 (3,4)	1,1–10,1	9 (8,2)	4,2 - 15,1	
Município de Residência* ( *n* *=* *273* )						0,189 a
Florianópolis	95 (34,8)	38 (30,7)	23,1–39,4	57 (38,3)	30,7–46,4	
Outros ^‡^	178 (65,2)	86 (69,4)	60,6 - 76,9	92 (61,7)	53,6–69,3	

Abreviações: IC95%: intervalo de confiança de 95%; HIJG: Hospital Infantil Joana de Gusmão; SINAN: Sistema de Informação de Agravos de Notificação.

a Teste qui quadrado.

b Teste Exato de Fisher.

*Dados sem informação de todos os registros.

†Estratificação segundo Marcondes. 29

‡ Agrupamento de todos os municípios brasileiros que foram citados como de residência na vítima.

A distribuição por sexo dos autores da agressão em relação ao sexo da vítima foi estatisticamente significante ( *p* < 0,05). A autoria dessas violências em 57,4% vinculou-se com ao menos 2 suspeitos que agiram conjuntamente, majoritariamente genitores (50,2%), sem suspeita do uso de álcool em 91,3% das notificações. Predominaram conhecidos (96,0%) ( Tabela 2 ).

**Tabela 2:** Caracterização dos autores dos maus-tratos (segundo sexo do autor, vínculo com a vítima, vínculo dicotomizado, número de autores e uso de ácool pelo autor), de acordo com o sexo das vítimas, SINAN-HIJG, 2016–2020 ( *n* = *276* )

Variáveis	Total	Feminino		Masculino		*p* -valor a
	n (%)	n (%)	IC95%	n (%)	IC95%	
**Sexo** * ( *n* *=* *248)*						**0,001** a
Masculino	55(22,2)	18 (15,5)	9,9–23,4	37 (28,0)	21,0– 36,4	
Feminino	55(22,2)	37 (31,9)	24,0–41,0	18 (13,6)	8,7–20,7	
Autores de ambos os sexos envolvidos na agressão	138 (55,7)	61 (52,6)	43,3–61,6	77 (58,3)	49,6– 66,5	
Vínculo com a vítima* ( *n* *=* *251* )						0,865 b
Mãe	39 (15,5)	22 (18,5)	12,4–26,6	17 (12,8)	8,1 - 19,8	
Pai	25 (10,0)	12 (10,1)	5,7 - 17,0	13 (9,9)	5,8 - 16,3	
Ambos †	126 (50,2)	56 (47,1)	38,2–56,2	70 (53,0)	44,3 - 61,5	
Mãe + outros ^‡^	13 (5,18)	7 (5,8)	2,8 - 11,9	6 (4,6)	2,0 - 9,8	
Pai + outros ^‡^	1 (0,40)	0 (0)	0 -0	1 (0,8)	0,1 - 5,3	
Própria pessoa	14 (5,58)	8 (6,7)	3,4–13,0	6 (4,6)	2,0 - 9,8	
Desconhecido	10 (3,98)	4 (3,4)	1,2 - 8,7	6 (4,6)	2,0 - 9,8	
Conhecido ou Familiar	22 (8,76)	10 (8,4)	4,5 - 15,0	12 (9,1)	5,2 - 15,4	
Pai + Mãe + Outros	1 (0,40)	0 (0)	0 -0	1 (0,8)	0,1–5,3	
**Vínculo com a vítima** dicotomizado* (*n* *=* *251* )						0,752 b
Conhecido	241 (96,0)	115(96,4)	91,3–98,8	126 (95,5)	90,2– 98,0	
Desconhecido	10 (4,0)	4 (3,4)	1,2–8,7	6 (4,6)	2,0–9,8	
Número de Autores* ( *n* *=* *251* )						0,403 a
1	107 (42,6)	54 (45,4)	36,6–54,5	53 (40,2)	32,0– 48,8	
2 ou mais	144 (57,4)	65 (54,6)	45,5–63,4	79 (59,8)	51,2– 68,0	
Uso de álcool* ( *n* *=* *161* )						0,263 b
Não	147 (91,3)	67 (88,2)	78,5–93,8	80 (94,1)	86,4–97,6	
Sim	14 (8,7)	9 (11,8)	6,1–21,5	5 (5,9)	2,4–13,6	

Abreviações : IC95%, intervalo de confiança de 95%; HIJG, Hospital Infantil Joana de Gusmão; SINAN, Sistema de Informação de Agraos de Notificação.

a Teste qui quadrado.

b Teste Exato de Fisher.

*Dados sem informação de todos os registros.

† Agrupamento dos vínculos pai e mãe.

‡ Agrupamento de todos os outros vínculos diferentes de “Pai” ou “Mãe,” mas que estejam associados a um deles.

A negligência foi a tipologia mais descrita (53,9%), o espancamento foi o meio mais presente (33,9%), resultando em fraturas em 31,5% dos casos, o eixo axial foi o segmento anatômico mais acometido (40,0%). Em relação entre local de fratura e sexo da vítima, as fraturas somente no esqueleto axial foram frequentes no sexo masculino e, fraturas em 2 ou mais segmentos predominaram no feminino ( *p* < 0,05). Quinze delas (17,6%) necessitaram intervenção cirúrgica. Houve cinco óbitos: 2 por ferimento por arma de fogo, 1 por espancamento, 1 por acidente automobilístico, 1 por queda de nível ( Tabela 3 ).

**Tabela 3:** Tipologia dos casos de maus tratos, suas consequências e desfecho “fraturas ósseas,” de acordo com o sexo das vítimas (segundo o tipo de violência, o meio, a presença de fraturas, o tipo de fratura, se houve consulta com ortopedista e se houve necessidade de cirurgia ou mortes), SINAN-HIJG, 2016–2020 ( *n* = *276* )

Variáveis	Total	Feminino		Masculino		*p* -valor
	n (%)	n (%)	IC95%	n (%)	IC95%	
**Tipo** ( *n* *=* *271* )						0,352 **a**
Negligência	146 (53,9)	61 (50,0)	41,1 - 58,9	85 (57,0)	48,9 - 64,8	
Física	106 (39,1)	50 (41,0)	32,5–50,0	56 (37,6)	30,1 - 45,7	
Outros **	19 (7,0)	11 (9,0)	5,0–15,7	8 (5,4)	2,7–10,4	
**Meio/Instrumento** * ( *n* *=* *248* )						0,297 **b**
Espancamento	84 (33,9)	39 (34,8)	26,5 - 44,2	45 (33,1)	25,6 - 41,5	
Acidente de Trânsito	52 (21,0)	21 (18,8)	12,5 - 27,2	31 (22,8)	16,4 - 30,7	
Choque Elétrico	33 (13,3)	14 (12,5)	7,5 - 20,1	19 (14,0)	9,0 - 21,0	
Quedas	32 (12,9)	20 (17,9)	11,7 - 26,2	12 (8,8)	5,0 - 15,0	
Arma de Fogo	17 (6,9)	5 (4,5)	1,8 - 10,4	12 (8,8)	5,0 - 15,0	
Outros***	30 (12,1)	13 (11,6)	6,8 - 19,1	17 (12,5)	7,9 - 19,3	
**Presença de Fraturas** * ( *n* *=* *270* )						0,537 **a**
Não	185 (68,5)	88 (70,4)	61,7 - 77,8	97 (66,9)	58,7 - 74,1	
Sim	85 (31,5)	37 (29,6)	22,2 - 38,3	48 (33,1)	25,8 - 41,2	
Tipo de Fratura* ( *n* *=* *85* )						** 0,017 a**
Somente Membro(s) Superior(es)	15 (17,7)	8 (21,6)	10,8–38,5	7 (14,6)	6,9–28,2	
Somente Membro(s) Inferior(es)	16 (18,8)	7 (18,9)	8,9–35,6	9 (18,8)	9,8–32,9	
Somente Esqueleto Axial	34 (40,0)	8 (21,6)	10,8–38,5	26 (54,17)	39,6–68,0	
Em 2 ou mais segmentos	20 (23,5)	14 (37,8)	23,3–54,99	6 (12,5)	5,5–25,8	
**Consulta ortopédica** *(n = 276)*						** 0,024 a**
Não	185 (67,0)	75 (60,0)	51,1–68,3	110 (72,9)	65.1–79,4	
Sim	91 (33,0)	50 (40,0)	31,7–48,9	41 (27,2)	21,0–34,9	
**Cirurgia** *( *n* *=* *276* )						0,912 **a**
Não	261 (94,6)	118 (94,4)	88,6 - 97,3	143 (94,7)	89,7 - 97,3	
Sim	15 (5,4)	7 (5,6)	2,7 - 11,4	8 (5,3)	2,7 - 10,3	
**Morte** *( *n* *=* *270* )						0,380 **b**
Não	265 (98,1)	122 (99,2)	94,3 - 99,9	143 (97,3)	92,9 - 99,0	
Sim	5 (1,9)	1 (0,8)	0,1–6,0	4 (2,7)	1,0 - 7,1	

IC95% = intervalo de confiança de 95%;

a Teste qui quadrado.

b Teste Exato de Fisher.

*Dados sem informação de todos os registros; NA: não aplicável.

**: Violência moral, maus-tratos, tentativa de suicídio.

***: Tentativa de suicídio, objeto contundente, objeto pérfuro-cortante, ameaça, enforcmento, intoxicação exógena, evasão, “shaken baby,” objeto quente.

HIJG: Hospital Infantil Joana de Gusmão; MI ou MMII: membro inferior/membros inferiores; MS ou MMSS: membro superior/membros superiores; SINAN: Sistema de Informação de Agravos de Notificação.

Na análise ajustada para sexo e idade, idade da vítima (menor de dois anos) (RC 2,48; IC 95%: 1,45 - 4,25), envolvimento de dois ou mais agressores (RC 2,09; IC 95%: 1,16 - 3,75), o meio ser trânsito/acidente automobilístico, (RC 2,65; IC 95%: 1,04–6,75), presença de consulta com ortopedista (RC 6,77; IC 95%: 3,66–12,51), e necessidade de intervenção cirúrgica (RC 36,72; IC 95%: 8,22–164,03) apresentaram associação estatisticamente significante com maior risco de fraturas ( Tabela 4 ).

**Tabela 4:** Fatores associados ao desfecho “Fraturas ósseas” nas vítimas de maus-tratos, SINAN-HIJG, 2016–2020 ( *n* = *270* ).

	Não ajustada		Ajustada p/ idade e sexo	
Variáveis	RC (IC95%)	*p* -valor	RC (IC95%)	*p* -valor
**Sexo da Vítima** *(n = 270)*				
Feminino (125)	1	0,537	1	
Masculino (145)	1,18 (0,70–1,97)		1,34 (0,79–2,29)	0,283
**Idade da vítima** *(n = 270)*				
≥ 2 anos (82)	1		1	
< 2 anos (188)	2,37 (1,39–4,03)	**0,001**	2,48 (1,45–4,25)	**0,001**
**Etnia** *(n = 270)*				
Não branco (41)	1		1	
Branco (239)	1,52 (0,62–3,71)	0,356	1,33 (0,53–3,31)	0,542
**Deficiência** *(n = 195)*				
Não (186)	1		1	
Sim (9)	0,66 (0,13–3,29)	0,616	0,74 (0,14–3,81)	0,718
**Município** ( *n = 267* )				
Florianópolis (94)	1		1	
Outros** (173)	0,33 (0,44–1,32)	0,325	0,78 (0,44–1,38)	0,395
**Sexo do(s) autor(es)** * *(n = 242)*				
Feminino (120)	1		1	
Masculino (122)	1,17 (0,67–2,03)	0,589	1,20 (0,66–2,17)	0,554
**Sexo do(s) autor(es)** * *(n = 242)*				
Feminino (53)	1		1	
Ambos (135)	1,64 (0,80–3,37)	0,176	1,68 (0,80–3,52)	0,169
Masculino (54)	0,79 (0,32–1,96)	0,607	0,87 (0,34–2,25)	0,775
**Número de Autores** *(n = 251)*				
1 agressor (107)	1		1	
Mais 2 agressores (144)	2,14 (1,19–3,83)	**0,011**	2,09 (1,16–3,75)	**0,014**
**Uso de álcool pelo autor** ( *n= 161* )				
Não (147)	1		1	
Sim (14)	0,99 (0,29–3,33)	0,988	1,26 (0,36–4,40)	0,714
**Vínculo com a vítima** *(n = 246)*				
Desconhecido (10)	1		1	
Conhecido (236)	1,72 (0,36–8,31)	0,499	1,61 (0,33–7,83)	0,554
**Tipologia** *(n = 265)*				
Outras (18)	1		1	
Negligência (145)	3, 26 (0,72–14,81)	0,126	2,60 (0,56–12,02)	0,221
Física (102)	4,95 (1,08–22,72)	**0,040**	3,67 (0,78–17,16)	0,099
**Meio** ( *n = 248* )				
*** Outros (53)	1		1	
Espancamento (79)	2,07 (0,87–4,92)	0,099	1,90 (0,78–4,60)	0,157
Trânsito (51)	2,51 (0,99–6,30)	0,051	2,65 (1,04–6,75)	0,042
Choque (33)	0,13 (0,16–1,10)	0,061	0,13 (0,15–1,07)	0,057
Queda nível (32)	2,21 (0,79–6,19)	0,131	2,28 (0,78–6,57)	0,129
**Consulta ortopédica** *(n = 270)*				
Não (179)	1		1	
Sim (91)	5,02 (2,88–8,73)	**<0,001**	6,77 (3,66–12,51)	**<0,001**
**Intervenção cirúrgica** *(n = 270)*				
Não (255)	1		1	
Sim (15)	16.52 (3.63–75,05)	**<0,001**	36,72 (8,22–164,03)	**<0,001**
**Evolução: morte** ( *n= 266* )				
Não (261)	1		1	
Sim (5)	1,46 (0,24–8,88)	0,684	1,09 (0,17–6,95)	0,928

Abreviações: IC95%,= intervalo de confiança de 95%; RC, razão de chance; SC: Santa Catarina; SINAN: Sistema de Informação de Agravos de Notificação.

*Dados sem informação de todos os registros.

**: Municípios que não são Florianópolis.

***: Tentativa de suicídio, objeto contundente, objeto pérfuro-cortante, ameaça, enforcmento, intoxicação exógena, evasão, “shaken baby,” objeto quente.

## Discussão

Vítimas do sexo masculino, como observado na literatura, 12 foram as mais acometidas (54,7%), com risco de 1,2 vezes de apresentar fraturas associadas, quando comparados ao sexo feminino.

Os extremos de idade (< 2 anos e > 10 anos) foram os grupos mais propensos a sofrer agressões. Os lactentes (< 2 anos), apresentaram um risco 2,4 vezes maior de presença de fraturas, quando comparados aos maiores de dois anos, independente do sexo, corroborando com a literatura internacional 6 7 19 e diferindo do computado pelo Disque 100, onde vítimas escolares foram as mais listadas. 9 Ressalta-se que quanto menor, mais dependente de cuidados a criança é, inclusive para chegar a um serviço de saúde, com subnotificação por omissão de cuidados. 2

Na região Sul do país predomina etnia branca, 8 26 explicando a disparidade entre notificações de violência em pessoas dessa etnia (89,4%) em relação às demais, diferente dos dados do Disque 100, que apontam a população de cor parda como a mais acometida por maus-tratos, seguida da branca e da negra. 9 Nesse estudo, possuir cor da pele branca relacionou-se a um risco 1,5 vezes maior para fraturas relacionadas aos maus-tratos, quando comparado às demais cores da pele.

A presença de vítimas portadoras de deficiência não foi expressiva (6,0%), divergindo da literatura, 1 2 7 9 15 19 levando a inferir que a não procura por atendimentos emergenciais para esses pacientes poderia relacionar-se a possíveis erros de notificação, à falta de diagnóstico da agressão e/ou de notificação e à incapacidade de verbalização de vítimas com deficiência. 2 26 Nesse grupo não houve associação com risco de fraturas.

O maior número de notificações de outros municípios (65,2%) que não a capital, pode justificar-se pelo hospital ser de grande porte e referência em ortopedia do estado.

Em relação à autoria dos maus-tratos, a maioria teve ao menos 2 agressores, principalmente pai e mãe simultaneamente, corroborando à literatura, onde mantém-se o padrão dos genitores como principais suspeitos dos maus-tratos infantis. 1 2 3 4 5 6 7 9 15 17 19 Em 91,0% dos casos os autores das agressões não estavam sob suspeita de uso de álcool, em consonância com a literatura, 2 9 15 não havendo relação entre o uso de álcool pelos agressores com o desfecho fraturas.

Corroborando pesquisas nacionais e internacionais, 2 3 4 5 6 12 a negligência (53,9%), seguida por agressões físicas (39,1%), foram as tipologias mais prevalentes, com distribuição semelhante entre os sexos. A alta prevalência desta última pode relacionar-se ao uso da força física, como forma de “educação,” prática disciplinadora, 1 2 12 onde pais que foram educados por meio do castigo e da punição física perpetram esse hábito culturalmente aceito, 1 2 independentemente da existência de políticas públicas, como a Lei n° 13.010 - “Lei do Menino Bernardo” 27 ou o Estatuto da Criança e do Adolescente. 28

Alerta-se para o risco aumentado dessas duas formas de maus-tratos na ocorrência de fraturas, que foi de 3,3 e 5,0 vezes maior, respectivamente, quando comparadas a todos os outros estudados. Depreendendo a importância da vigilância dos pais aos seus filhos e principalmente da educação continuada sobre os acidentes e lesões não intencionais. 2 3 7

Quando avaliados os meios da agressão, “acidentes de trânsito” e “espancamento” responderam a mais da metade das notificações (54,9%), seguidas menos expressivamente por choque elétrico (13,3%) quedas de nível (12,9%) e “outras,” semelhante à literatura, 2 6 7 9 19 sinalizando para a prática da força física como medida disciplinadora educativa 1 2 12 e a ausência da observância do transporte seguro. 2 29 “Espancamento” e “acidentes de trânsito,” relacionaram-se respectivamente a um risco de 2,1 e 2,5 vezes de ocasionar fraturas.

O desfecho “fraturas” foi 2,2 vezes mais frequentemente observado nas “quedas de nível,” atentando à necessidade da vigilância e supervisão, especialmente dos menores, que estão iniciando a deambulação. 2 3 7 Sua prevalência (31,5%) foi 4,5 vezes maior do encontrado em outro estudo nacional. 15

Quanto à localização anatômica das fraturas, 40,0% estavam presentes somente no esqueleto axial - incluindo crânio, região relacionada a desfecho de maior gravidade. 2 3 4 5 6 7 12 15 18 19 23,5% das vítimas apresentaram lesões em mais de um segmento, sendo fator de risco de maus-tratos 2 3 4 5 6 7 11 18 19 , ou seja, uma criança com múltiplas fraturas, principalmente em mais de um local anatômico, deve ser avaliada mais atentamente para esta suspeição diagnóstica.

Dos pacientes estudados, independente do sexo, os menores de 2 anos tiveram um risco 2,5 vezes maior de sofrerem fraturas do que os outros grupos de idade, além de 1,7 vezes mais risco para o mesmo desfecho se a violência fosse perpetrada por conhecidos, quando comparados aos que foram agredidos por desconhecidos. Outros fatores associados ao maior risco de fraturas foram: a agressão ser cometida por 2 autores ou mais, vítimas de acidentes automobilísticos e o atendimento realizado por ortopedista - riscos respectivamente 2,1; 2,7 e 6,8 vezes maiores. Considerando que acidentes automobilísticos se relacionam por vezes à negligência, depreende-se um grande desafio: transporte seguro de crianças estabelecido nas regras de segurança do transporte de crianças nos veículos. 29

Quinze pacientes (17,7%) foram submetidos a cirurgias ortopédicas como parte do tratamento e a presença de fratura óssea relacionou-se a 16,5 vezes mais necessidade de intervenção cirúrgica e a 1,5 vezes o risco de morte, quando comparada a ausência da mesma no estudo.

Esses dados reforçam a importância da prevenção, 2 29 da atenção aos sinais que podem levantar suspeitas de maus-tratos e da investigação adequada por parte do profissional da “linha de frente” e do encaminhamento ao especialista quando pertinente. 6 7 19

Cita-se como provável limitação a fonte secundária dos dados, dirimida por meio da checagem manual uma a uma das fichas de notificação e da conferência no prontuário hospitalar das vítimas.

## Conclusão

Pela dependência e vulnerabilidade inerentes ao ciclo de vida, as crianças são um grupo de risco para as diversas violências quer sejam acidentais ou intencionais, necessitando tanto a educação da família para a prevenção, como a atenção do profissional da assistência na identificação e correta notificação deste agravo, com condução adequada dos casos, evitando-se desfechos graves.
